# Transcriptional and post-transcriptional regulation of retrotransposons IAP and MuERV-L affect pluripotency of mice ES cells

**DOI:** 10.1186/1477-7827-4-55

**Published:** 2006-11-08

**Authors:** Miguel A Ramírez, Eva Pericuesta, Raul Fernandez-Gonzalez, Pedro Moreira, Belen Pintado, Alfonso Gutierrez-Adan

**Affiliations:** 1 Departamento de Reproducción Animal, INIA, Ctra. De La Coruña Km 5,9, Madrid 28040, España

## Abstract

**Background:**

In the mouse, culture of embryonic stem (ES) cells may decrease their pluripotency and give rise to foetal abnormalities in recipient embryos. These abnormalities are frequently associated with both, chromosome abnormalities or epigenetic alteration of imprinting genes; however, little is known about the epigenetic stability of endogenous retrotransposable elements (REs). In our laboratory, we came across a R1 ES cell line, which at passage 27, lost the ability of germline transmission and started inducing the kinky tail phenotype in all chimeric animals produced with it.

**Methods:**

In order to investigate whether this phenotype was associated with chromosome alteration, inadvertent differentiation, or epigenetic modification, we characterized and compared this R1 ES cell line at passage 27 with an early passage and with a second ES cell line C57/CBAF1 generated in our laboratory. We assessed: i) karyotype; ii) expression of pluripotent and differentiation markers, iii) mRNA transcription by qRT-PCR of two REs, intracisternal-A particle (IAP) and murine endogenous-retrovirus-L (MuERV-L), and iv) methylation of IAP and MuERV-L.

**Results:**

The R1 ES cell at passage 27, presented normal morphology, karyotype, and expression of genetic markers characteristic of pluripotent; however, it was detected an altered mRNA transcription of sense and antisense RNA strands of both REs, concomitantly with an altered methylation pattern for the IAP element but not for MuERV-L. These results indicate that besides methylation, other post-transcriptional processes are involved in gene silencing of some REs; and that culture of ES cells may decrease their pluripotency by producing inadvertent alterations in the expression of REs without significantly affecting the morphology, chromosome structure, and expression of pluripotent or differentiation markers.

**Conclusion:**

Inadvertent REs instability may have important consequences for the use of ES cells in transgenesis (chimera formation) or in cell therapy.

## Background

It is generally accepted that an early-passage ES cell line can be used to produce complete ES cell-derived foetuses [1]. However, upon prolonged culturing, the genetic and/or epigenetic potential of the majority of these ES cell lines becomes limited. Foetuses completely derived, or with a strong contribution of these high passage stem cells, may suffer from several developmental problems, such as, increased size and body mass, polydactyly, swollen oedematous skin, and perinatal death [1-3]. It has been hypothesized that such developmental problems result from the accumulation of chromosome abnormalities and/or from epigenetic alterations in contributing ES cells [2]. ES cell gene expression is modulated by the epigenetic regulation of its genome; and such regulation, is sensitive to culture environment influences and characterized by particular chromatin modifications [4]. Such chromatin modifications may include the methylation, acetylation, ubiquitination and phosphorylation of the amino terminal tail of core histones, or the direct methylation of the DNA itself [5]. The most described chromatin modification is DNA methylation, which is mainly characterized by the covalent addition of a methyl group at the position 5 of cytosine residues in CpG dinucleotides. DNA methylation is a key factor in the control mechanism of gene expression and epigenetic regulation [6]. After fertilization, most methylation marks, with the exception of the ones associated with the imprinting established during gametogenesis, are removed from the embryonic genome to be gradually reset until blastocyst stage [7]. The epigenetic reprogramming of most genes is however, not complete until after implantation [8]. Recently, it has been reported that retrotransposon elements (RE) are transcribed during early mouse embryogenesis [9] and in ES cell lines [10]; and that the expression of REs also regulates host genes in preimplantation embryos [9]. Since ES cells are usually isolated from blastocysts, the expression of REs may be essential for preservation of the genomic integrity and pluripotency of these cells. In humans, it has been reported that retrotransposons can have an effect on cell differentiation [11].

Transposable elements (TE) span across major segments of the eukaryotic genome, representing, for example, more than 40% of mouse sequences. In mammals, almost all TE fall into one of four types, of which three transpose through RNA intermediates and one transposes directly as DNA. These are long interspersed elements (LINEs), shortinterspersed elements (SINEs), LTR REs and DNA transposons. REs replicate by transcription of an RNA intermediate, subsequent reverse transcription, and insertion of a new copy into a new location in the genome. Although a variety of LTR REs exists, only the vertebrate-specific endogenous retrovirus-like (ERV) appear to be active in the mammalian genome. These are characterized by flanking long terminal repeats (LTRs) which regulate the transcription of internal viral genes. There are three classes (I-III) of active ERVs in mice, and intracisternal-A particles (IAPs) are the most abundant of the active class II elements. IAPs are expressed during early embryo development [9]. Increased IAP expression correlates with decreased DNA methylation commencing around the 8-cell stage, and de novo DNA methylation that occurs following blastocysts formation in coupled to the repression of IAP expression [12]. We have decided to characterize the epigenetic regulation of these elements in our ES cell lines since they are one of the most aggressive parasitic sequences known in the mouse genome, which could be responsible for the phenotype observed. Also, because there are multiple examples in the literature where IAPs become associated with metastable epialleles [13], which represent a distinct and novel group of epigenetically-sensitive genes that display variegation, variable expression in genetically identical individuals and transgenerational epigenetic inheritance. In addition, in some respects IAPs are similar to imprinted genes, because IAP elements are considerably resistant to the epigenetic reprogramming occurring during preimplantation [14]. The resistance to demethylation of IAPs might be beneficial to the host organism since many of these elements are capable of retrotransposition, which would have detrimental consequences in the form of mutations [14]. In our study, we have also analyzed MuERV-L, a recently discovered ERV element of the class III REs. We have selected this RE specifically, because several studies concluded that it is still an active element in the mouse genome, capable of generating unexpected phenotypes apparently due to its sequence conservation and intact open reading frames (ORFs) [15,16], capable of generating unexpected phenotypes. MuERV-L is one of the earliest transcribed genes in mouse 1-cell embryos [17], that is highly transcribed in 2-cells embryos but poorly transcribed at blastocyst stage [9]. Moreover, it has been observed that these two retrotransposons have both sense and antisense RNA expression at blastocyst stage and that at this early stage of development an RNA interference (RNAi)-mediated post-transcriptional mechanism constrains expression of these REs [18].

In this study, we have analyzed the genetic and epigenetic status of an ES cell line that at passage 27 lost the ability of germline transmission and started inducing the kinky tail phenotype in all chimeras produced with it. Although many other parameters were analysed, this ES cell line was only significantly different relatively to others in its sense and antisense mRNA expression pattern and methylation profile of repetitive parasitic sequences. Then, we investigated if the differences in RE expression were due to a transcriptional mechanism of gene silencing (methylation) or to a process that acts at the level of expressed transcripts. We concluded that inadvertent REs instability would have important consequences for the use of ES cells in transgenesis (chimera formation) or in cell therapy.

## Methods

### Reagents and Media

All chemicals and media were purchased from Sigma Chemical Co. (Madrid, Spain) unless otherwise stated.

### ES cell culture, embryoid bodies and chimera production

Standard methods for maintaining and differentiating ES cells and for chimera production have been described in detail elsewhere [19]. Briefly, R1 ES cells (from A. Nagy laboratory, with a 129/Sv × 129/Sv-CP F1 background) and MAR1 ES cells (generated in our laboratory with a C57 × CBA F1 genetic background) were grown on mitomycin C-treated mouse embryonic fibroblast (MEF) feeder layers plated on 0.1% vol/vol gelatine-coated tissue plates, and maintained with Dulbecco's Modified Eagle Medium (DMEM; Invitrogen, Carlsbad, CA, USA) supplemented with 20% FBS (PAA Laboratories Cölbe Germany), 2 mM glutamine, 1 mM MEM nonessential amino acids solution, 1 mM β-mercaptoethanol, 1000 U/ml leukaemia inhibitory factor (LIF), 100 U/ml penicillin and 100 μg/ml Streptomycin.

To produce embryoid bodies (EBs), ES cells were trypsinized and back-plated for 30 minutes to deplete fibroblasts, and then plated in non-adherent 10 cm bacterial-grade Petri dishes (5 × 10^5 ^cells per dish) in ES medium without LIF. Embryoid bodies were collected after six days using a mouth controlled finely plugged Pasteur pipettes, and were used for mRNA analysis.

For chimera formation, 10–15 ES cells were injected into the blastocoele cavity of 3.5 dpc blastocysts of CD1 mice, in a microinjection drop containing M2 medium using a Piezo-driven injector (PMM150FU, Prime Tech, Ibaraki, Japan) and Eppendorf micromanipulators (Eppendorf TransferMan NK 2, Hamburg, Germany) attached to an inverted microscope. The blastocysts were returned to the oviducts of 0.5 dpc pseudopregnant CD1 foster mothers on the day of microinjection after 1 hour recovery in KSOM medium previously equilibrated at 37°C in a 5% CO_2 _air atmosphere.

### Karyotype analysis

Chromosome spreads of the ES cell lines were performed as described below. ES cells were arrested in metaphase by supplementing the culture medium with 0.1 μg/ml colcemid for 2 hr at 37°C in a 5% CO_2 _air atmosphere; after that, cells were treated with trypsin-EDTA for 2 min at 37°C. After pipeting, the single cell suspension was washed twice with PBS by centrifugation at 200 G for 5 min. The pellet obtained was exposed to a hypotonic stock by resuspension in 0.075 M KCl for 15 min at 37°C. After a second centrifugation step the hypotonic solution was removed, and the pellet fixed with a methanol/acetic acid solution (3:1; vol/vol) by gently pipetting. Ten min later, cells were pelleted again and fixed a second time. Before slide mounting, cells were washed twice with PBS. The slides were dried overnight at 55°C, stained in freshly made 10% Giemsa solution for 30 min, and rinsed with distilled water. Finally, air-dried slides were observed with an Optiphot II microscope (Nikon, Tokyo, Japan) with a magnification of 1000 ×.

### Analysis of marker gene expression by RT-PCR

Total RNA was extracted from ES cell pellets using the Ultraspect™ RNA Isolation System (Biotecx Lab. Inc., Houston, Texas, USA) following the manufacture's instructions. The precipitated RNA was dissolved in DEPC-treated water, and was digested with 2U of DNAse I (TURBO DNA-free™, Ambion, Austin, TX, USA) at 37°C for 20 min, to ensure that the only source of DNA in the polymerase chain reaction (PCR) was cDNA from cellular RNA. Finally, the RNA was extracted with phenol purification and ethanol precipitation, reconstituted with 50 μl of DEPC-treated water, and stored at -80°C until the RT-PCR. The RT reaction was carried out following the manufacturer's instructions (Epicentre Tech. Corp., Madison, Wisconsin). RNA were dissolved in water, heat-denatured at 65°C for 2 min, and reverse-transcribed at 37°C for 60 min in a final volume of 25 μl containing 0.5 mM of each dNTP, 0.2 μM oligo (dT), 0.5 μl MMLV-RT, 0.2 μl RNAsin, 1 × MMLV-RT buffer and 8 mM DTT. After reverse transcription, the PCR amplification of the different genes was performed by adding 1 μl of each sample to the PCR mix containing the specific primers. The PCR products were subjected to electrophoresis in a 1,5% agarose gel. Table 1 lists the primers used for the RT-PCR. GAPDH was used as positive control for this RT reaction, while negative control experiments were done in the absence of template RNA, and the absence of genomic contamination was systematically checked with GAPDH amplification of the RNA samples without reverse transcriptase. Generation of expected fragments was strictly dependent on the presence of RNA in the RT reaction. Genes previously reported as markers of early differentiation into germ layers or into tissue-specific precursors, were chosen as sensitive indicators of differentiation. Genes previously reported to be associated with a pluripotent state in blastocysts and ES cell populations were chosen as sensitive indicators of pluripotency.

**Table 1:** Primers used for the RT-PCR of genes commonly expressed in differentiated and undifferentiated ES cells.

Gene	Primer 5'-3' (Forward/Reverse)	Size	UniGene
GATA-4	GCCTGTATGTAATGCCTGCG/CCGAGCAGGAATTTGAAGAGG	469	Mm. 247669
GATA-2	ACCCACGCCACCCAAAGAAGTG/GCCGCCTTCCATCTTCATGCTC	157	Mm. 272747
AFP	TTTTCTGAGGGATGAAACCTATG/AAGCTCTTGTTTCATGGTCTGTA	116	Mm. 358570
Msx-1	GCTATGACTTCTTTGCCACTCG/TTAAGAGAAGGGGACCAGGTGG	1016	Mn. 259122
Brachyury	GCTGTGACTGCCTACCAGCAGAATG/GAGAGAGAGCGAGCCTCCAAAC	220	Mn. 913
Myf5	TGCCATCCGCTACATTGAGAG/CCGGGGTAGCAGGCTGTGAGTTG	352	Mn. 4984
Keratin-15	CACCACATTCTTGCAAAC/ATTAAGGTTCTGCATGGTC	313	Mn. 38498
HNF3-β	GGACGTAAAGGAAGGGACTCCAC/AGCCCATTTGAATAATCAGCTCAC	174	Mn. 938
Nestin	AGTGTGAAGGCAAAGATAGC/TCTGTCAGGATTGGGATGGG	316	Mn. 23742
Vimentin	AAGGGTGAGTAGAGAGTTC/AACACTGTTAGGAAAGAGG	222	Mn. 7
β3-tubulin	TCACTGTGCCTGAACTTACC/GGAACATAGCCGTAAACTGC	318	Mn. 40068
Nanog	AGGGTCTGCTACTGAGATGCTCTG/CAACCACTGGTTTTTCTGCCACCG	363	Mn. 6047
Oct3/4	GGAGAGGTGAAACCGTCCCTAGG/AGAGGAGGTTCCCTCTGAGTTGC	391	Mn. 17031
Rex1	CCAGGGAAGGATGAGAGA/TAGAAGCTGGTAACAGGGAG	264	Mn. 3396
GENESIS	TCTTACATCGCGCTCATCAC/TCTTGACGAAGCAGTCGTTG	171	Mn. 4758
FGFR-4	TCCGACAAGGATTTGGCAG/GCACTTCCGAGACTCCAGATAC	400	Mn. 4912
TERF1	TTCAACAACCGAACAAGTGTC/TCTCTTTCTCTTCCCCCTCC	215	Mn. 4306
Cx43	TACCACGCCACCACTGGCCCA/ATTCTGGTTGTCGTCGGGGAAATC	290	Mn. 4504
GLUT1	CAGTCAGCAATGAAGTCCAG/AGCAGTAAGTTCTCAGCCTC	585	Mn. 30044
BCRP1/ABCG1	CCATAGCCACAGGCCAAAGT/GGGCCACATGATTCTTCCAC	326	Mn.196728
GAPDH	GGGTGTGAACCACGAGAAATATGA/CCTTCCACAATGCCAAAGT	250	Mm.379644

### Quantification of sense, antisense, and total mRNA expression of IAP and MuERV by real-time PCR

**Figure 3:**
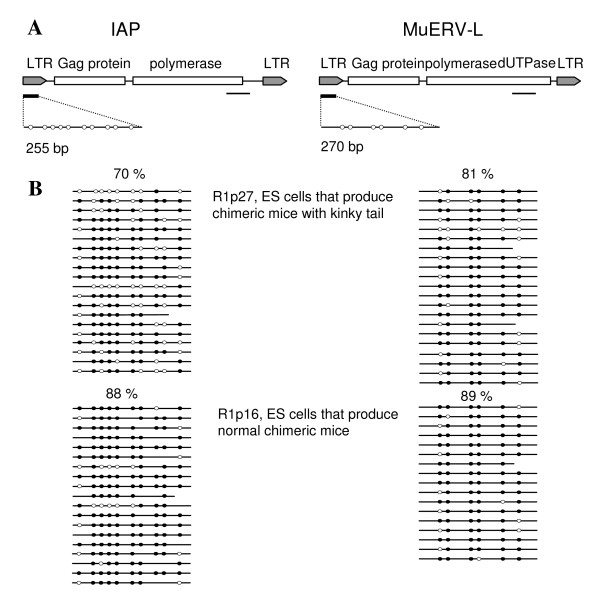
Methylation profiles of two retrotransposables elements, intracisternal-A particle (IAP) and murine endogenous retrovirus-L (MuERV-L) in R1p16 and R1p27 ES cells. (A) Regions analyzed by bisulphite sequencing in IAP and MuERV-L. LTR, long terminal repeat. Filled circles represent CpG dinucleotides present in the regions analysed. (B) Individual DNA methylation profile following bisulphite treatment and amplification of ES cell DNA. Methylated and unmethylated regions are represented by filled circles and open circles, respectively. The overall percentage of methylated CpG dinucleotides is shown above each group of clones.

RNA was extracted from ES cell as described before. Standard methods for extraction of RNA from blastocysts stage embryos have been described in detail elsewhere [20]. Briefly, total RNA was prepared from 6 groups of pools of 12 embryos, previously washed in PBS supplemented with 0.1% (w/v) polyvinyl pyrrolidone and was stored at -80°C until processed for RT-real time-PCR. RNA was extracted from each pool of embryos using the Strataprep Total RNA microprep kit (Stratagene, La Jolla, CA, USA) according to the manufacturer's instructions. Before the RT, total RNA purification was digested with DNase I. RNA from 4 blastocysts equivalents were used per PCR. Reverse-transcribed was realized as described before using the primers indicated in the Table 2. Absence of genomic contamination was systematically checked with IAP and MuERV amplification of the RNA samples without reverse transcriptase. For quantification of mRNA transcription of IAP and MuERV elements, RT was realized with random primers; and for the quantification of the sense and antisense mRNA expression of IAP and MuERV, RT was primed with either RTF1 or RTR2 primer specific for each element and the reverses-transcribed cDNA was amplified using the inner pair of PCRF3 and PCRR4 primers (Table 2; Relative position of primers are indicated in Fig. 3). The forward primer for the GAPDH internal control was also included in each RT reaction. Relative amount of transcript was quantified by real time quantitative RT-PCR (qRT-PCR) in three replicate PCR experiments. Details of the qRT-PCR are described previously [21]. Briefly, PCR was performed using a Rotorgene 2000 Real Time CyclerTM (Corbett Research, Sydney, Australia) and SYBR Green (Molecular Probes, Eugene, OR) as a double-stranded DNA-specific fluorescent dye. The PCR reaction mixture (25 μl) contained 2.5 μl 10 × buffer, 3 mM MgCl2, 2 U Taq Express (MWGAG Biotech, Ebersberg, Germany), 100 μM of each dNTPs, and 0.2 μM of each primer. In addition, the double-stranded DNA dye, SYBR Green I, (1:3000 of 10000 × stock solution) was included in each reaction. The PCR protocol included an initial step of 94°C (2 min), followed by 40 cycles of 94°C (15 sec), 56–59°C (30 sec) and 72°C (30 sec). Fluorescent data were acquired at 85°C. The melting protocol consisted of holding at 40°C for 60 sec and then heating from 50 to 94°C, holding at each temperature for 5 sec while monitoring fluorescence. Product identity was confirmed by ethidium-bromide-stained 2% agarose gel electrophoresis. In addition, amplicon identities were confirmed by sequencing of PCR products. As negative controls, tubes were always prepared in which RNA or reverse transcriptase was omitted during the RT-reaction.

**Table 2:** Primers used for the sense and antisense real time PCR (qRT-PCR) of IAP and MuERV-L.

Transposon	Primer 5'-3'	*Tm *(°C)
IAP-RTF1	TCAAGGACAGGGTATTGTTG	For reverse transcription
IAP-RTR2	TATTGACGCCCTGGACATCAC	For reverse transcription
IAP-PCRF3	GGGTATTGTTGAGCGTGCGC	56
IAP-PCRR4	TCGGGTGAGTCTTTCTGGTAC	56
MuERV-RTF1	CAAAGTGGCCATGGTGGTCG	For reverse transcription
MuERV-RTR2	GTACCATATATCGAGCGCTG	For reverse transcription
MuERV-PCRF3	TGCTTGGGCTCAGCAACATGG	56
MuERV-PCRR4	GACAGAATGCCTCATCTATCGT	56

The comparative CT method was used for quantification of expression levels [22] using GAPDH as endogenous control. Fluorescence was acquired in each cycle in order to determine the threshold cycle or the cycle during the log-linear phase of the reaction at which fluorescence increased above background for each sample. Within this region of the amplification curve, each difference of one cycle is equivalent to a doubling of the amplified product of the PCR. According to the comparative CT method, the ΔCT value was determined by subtracting the GAPDH CT value for each sample from each gene CT value of the sample. Calculation of ΔΔCT involved using the highest sample ΔCT value (i.e. the sample with the lower target expression) as an arbitrary constant to subtract from all other ΔCT sample values. Fold changes in the relative gene expression of target was determined by using the formula, 2-ΔΔCT.

Data on mRNA expression were analyzed using the SigmaStat (Jandel Scientific, San Rafael, CA) software package. One-way repeated-measures ANOVA (followed by multiple pair-wise comparisons using Student-Newman-Kleus method) were used for the analysis of differences in mRNA expression assayed by quantitative RT-PCR.

### Bisulphite analysis of mouse ES cells and tail tissue samples

DNA from ES cells and from tail tissue was extracted using standard proteinase K digestion and phenol-chloroform extraction methods. The isolated DNA was treated with sodium bisulphite using the EZ DNA Methylation Kit (Zymo Research, Orange, CA, USA). The bisulphite-modified DNA was amplified by PCR. The methylated status of IAP LTRs (accession M17551) was examined using the following primers: IAP-F1: 5'-TTGATAGTTGTGTTTTAAGTGGTAAATAAA; IAP-R1: 5'-CAAAAAAAACAC CACAAACCAAAAT; IAP-F2: 5'-TTGTGTTTTAAGTGGTAAATAAATAA TTTG; IAP-R2: 5'- AAAACACCACAAACCAAAATCTTCTAC. PCR conditions were: 1st PCR (30 cycles) F1/R1; 2nd PCR (30 cycles): F2/R2. Temperature conditions were: 94°C, 3 min; 94°C, 20 sec; 55°C, 30 sec (2nd PCR, 60°C); 72°C, 30 sec; 72°C, 5 min. The methylated status of MuERV-L LTRs (accession AC166650) was examined using the following primers: RVL-F1: 5'-GTTATTATGTGATTTGAATTA; RVL-R1: 5'-ACATACAAAACCATCAATAAAC; RVL-F2: 5'-TTTATTATGAGTTGGGTAT; RVL-R2: 5'-ATAAACCAAACTCTAATCTTC. PCR conditions were: 1st PCR (30 cycles) F1/R1; 2nd PCR (30 cycles): F2/R2. Temperature conditions were: 94°C 3 min, 94°C 20 sec, 53°C 30 sec (2nd PCR 60°C), 72°C 30 sec, 72°C 5 min. PCR products were gel-purified using the ELU-QUIK DNA purification kit (Schleicher&Schuell, Dassel, Germany) and transformed into XL1 *Escherichia coli *cells. Positive clones were verified by restriction analysis and the products were sequenced using standard methods.

The methylation percentages were obtained for each individual clone within a sample (number of methylated CpGs per clone divided by the total number of CpGs per clone). These were then used to calculate the overall methylation level and standard error of the mean of each sample. A logistical regression test from the SigmaStat statistical package was used to test for differences between samples. The samples are considered significantly different when P < 0.05.

## Results

### R1p27 chimeric mice display a kinky tail phenotype

**Figure 1:**
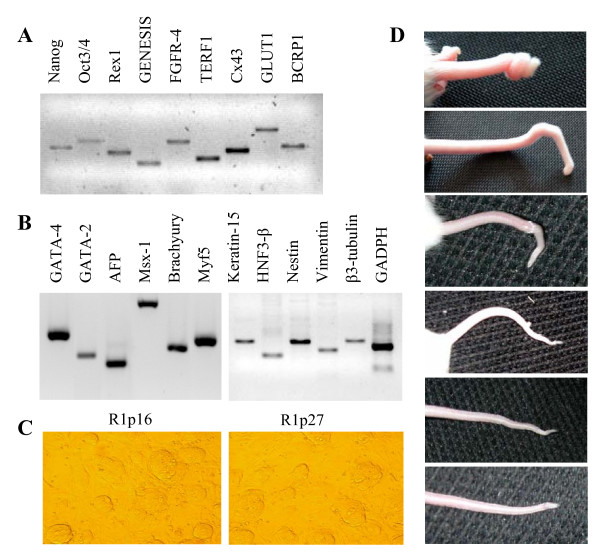
RT-PCR characterization of the undifferentiated state of R1p16 and R1p27 ES cells with commonly used genetic markers. The three cells group analyzed showed similar expression of the (A) genetic markers of pluripotency, and of the (B) genetic markers of differentiated phenotypes. (C) Morphological similarity between the R1p10 and the R1p27 ES cell line responsible for the kinky phenotype in our chimeras. (D) Examples of the variable kinky tail phenotype consistently observed in different chimeric mice produced with the R1p27 ES cell line.

In this study we have used an MAR1 ES cell line at passage 10 (C57 × CBA F1), and a R1 ES cell line at passage 16 and passage 27 (129/Sv × 129/Sv-CP F1), which we designated R1p16 and R1p27, respectively. No morphologic differences were observed between early or late passage of R1 ES cells (Fig. 1C). Three plates of at least three different thaw were used in every experiment, Forty-six chimeric animals were produced with R1p27 ES cells. Although, otherwise normal, all chimeric animals presented a characteristic, but variable, kinky tail phenotype (Figure 1D). This chimeric phenotype was very uncommon in our previous ES cell work experience. The level of chimerism produced with R1p27 ES cells was relatively low (in the range of 10 % to 50 %), never producing germ line transmission. Contrary to these results, none of the chimeric animals produced with MAR1 or R1p16 ES cells (more than 50 were generated) displayed any detectable abnormal phenotype. The level of chimerism of these animals was variable (ranging from 25% to 75%) and a reasonable proportion of them (10% to 20%) were capable of germ line transmission.

From the chromosome analysis performed on these ES cell lines, karyotype abnormalities were not detected. And for R1 this was independent of passage number. More than 80% of metaphase spreads prepared from MAR1, R1p16 and R1p27 ES cells displayed the appropriate species-specific chromosome number.

### Expression analysis of genetic markers characteristic of pluripotent or differentiated cells

The pluripotency state of the ES cells used in this study was evaluated by the expression analysis of several markers commonly associated with undifferentiated and differentiated ES cells [23,24]. Nanog [25,26], Oct3/4 [27] and Rex1 [28] were used as genetic markers of pluripotency. OCT3/4, SOX-2, GENESIS [29], FGFR-4 [30], and telomerase associated factor TERF1 [31] were used as undifferentiated markers. In addition, the expression of other markers present on blastocysts or other stem cell populations such as Cx43 [32], GLUT1 [33], and BCRP1/ABCG1 [34] were also examined. In order to assess the expression of genetic markers characteristic of differentiated tissues, we have used published RT-PCR primers that amplify genes characteristic of endoderm (GATA-4, GATA-2, and AFP), mesoderm (Msx-1, Brachyury, Myf5, Keratin-15, and HNF3-β) and ectoderm (Nestin, Vimentin, and β3-tubulin). Bands of the appropriate size were observed for all the genetic markers analysed using species-specific primers in MAR1, R1p16 and R1p27 ES cells (Table 1 and Fig. 1A, B). All pluripotent markers except Glut-1 were not detected in the feeder cells used for ES cell culture (data not shown).

RT-PCR analysis of the markers associated with undifferentiated and differentiated ES cells showed that R1p16 and MAR1p10 ES cells have the same pattern of expression (Table 3), and that this expression pattern is in agreement with previously reported pluripotent ES cell characterizations [24]. Moreover, in R1p27 ES cells only 4 of 21 markers have different expression profiles. Concomitantly with these results was the fact that differences in the morphology of the ES cells used in this study could not be detected. Differential qualitative expression in the R1p27 ES cells, was just observed for two of the 11 undifferentiated markers analysed (Rex1 and Genesis), and two of the 11 differentiation markers evaluated (HNF3-β and β3-tubulin) (Table 3). GATA 2, a zinc finger transcription factor that it has been detected also in mouse D3 ES cell line [24], was the only marker of differentiation present in R1p16 and MAR1p10 ES cells that was also detected in the R1p27 ES.

**Table 3:** mRNA expression of genetic markers characteristic of pluripotent and differentiated phenotypes.

	R1p16	MAR1p10	R1p27		R1p16	MAR1p10	R1p27
GATA-4	-	-	-	Nanog	+	+	+
GATA-2	+	+	+	Oct3/4	+	+	+
AFP	-	-	-	Rex1	+	+	-
Msx-1	-	-	-	GENESIS	+	+	-
Brachyury	-	-	-	FGFR-4	+	+	+
Myf5	-	-	-	TERF1	+	+	+
Keratin-15	-	-	-	Cx43	+	+	+
HNF3-β	-	-	+	GLUT1	+	+	+
Nestin	-	-	-	BCRP1	+	+	+
Vimentin	-	-	-	GAPDH	+	+	+
B3-tubulin	-	-	+				

### Sense and antisense RNA expression profile of IAP and MuERV-L retrotransposable elements in ES cells at early or late passages

**Figure 2:**
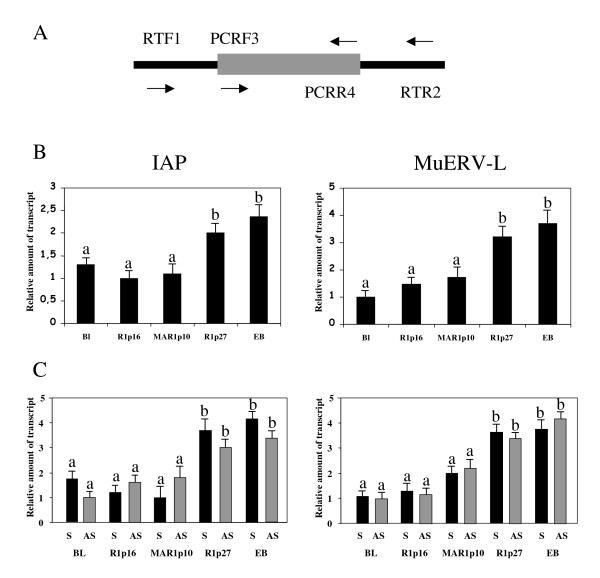
Quantitative RT-PCR analysis of IAP and MuERV-L. (A) Position of primers used in the experiment. (B) Pattern of IAP and MuERV-L mRNA expression. Each lane represents the mean plus standard desviation of 3 technical replicates from 3 independent prepared samples, expressed relative to the group in which the expression was the lowest. The region used for the qRT-PCR expression analysis of these elements is indicated with a line at the 3'end of each element as shown in Fig 3A. BL, blastocyst. (C) Quantitative RT-PCR of sense and antisense RNA strands from IAP and MuERV-L. Each lane represents the results of 3 replicates from 3 independent prepared samples, expressed relative to the group in which the expression was the lowest. a, b: refers to significant differences in relative transcript abundance between column (P < 0.05).

Both REs exhibited increased expression in R1p27, similar to that observed in embryo bodies generated from R1p16 (Fig 2B). In R1p27, the sense and antisense expression of these elements was higher than in other ES cells analyzed, but similar to that detected in EB (Fig 2B, C). Moreover, after cDNA amplification, the expression of the IAP sense element in R1p27 was three time higher than in R1p16 or MARp10, but the expression of the IAP antisense element was less than double than in R1p16 or MARp10, indicating an apparent alteration of the expression balance between sense and antisense RNA strands in this ES cell line. Such modification in both sense and antisense RNA expression of the two REs analysed suggests an important role for these repetitive sequences in ES cells.

### Methylation patterns of IAP and MuERV-L retrotransposons in R1p27 ES cells

For the bisulphite analysis of the methylation pattern of the retrotransposon IAP, primers were designed against its 5'LTR sequence in order to amplify a 255 bp fragment containing 10 CpG dinucleotides spanning the IAP promoter (Fig. 3A), known to be methylation sensitive [14]. A similar strategy was followed for the 5'LTR sequence of the MuERV-L retrotransposon allowing the amplification of a 270 bp fragment containing 6 CpG dinucleotides spanning its promoter [9] (Fig. 3A). To determine is the samples were completely converted by the bisulphite treatment, we amplify the bisulphite treated DNA with primers specific for untreated DNA (IAP-PCRF3, IAP-PCRR4, MuERV-PCRF3, and MuERV-PCRR4; Table 2). Using these primers we could not amplify any sample, confirming that our bisulphite treatment give a complete conversion of unmethylated cytosines. Then, we analyzed the methylation profiles of IAP and MuERV-L repeated sequences in bisulphite treatment DNA samples obtained from the R1p16, R1p27, and MARp10 ES cells, and from the kinky tail of 10 chimeric mice generated with the R1p27 ES cells. Whereas no significant methylation differences in the promoter region of the MuERV-L transposable element were observed between ES cell types, the number of CpG dinucleotides methylated in the IAP promoter of R1p27 ES cells was significantly lower than the one observed in R1p16 or MAR p10 ES cells (P < 0.05; Fig. 3B). No differences were found in the methylation profile of the tails (data not shown) probably indicating the lower level of chimerism produced with the R1p27 ES cells.

## Discussion

Although ES cells are believed to divide infinitely by self-renewal division, there is no evidence that demonstrates their infinite replicative ability. ES cell pluripotency is modulated by the genetic and epigenetic regulation of its genome. During prolonged culture, the genetic and/or epigenetic potential of the majority of the ES cell lines can be altered. Recently it has been reported that human ES cells lines maintained in vitro can develop epigenetic alterations [35]. In this study, we describe alterations in the epigenetic status of an ES cell line that at passage 27 lost the ability of germline transmission and started inducing the kinky tail phenotype in all chimeras produced with it. We observed for the first time that culture of ES cells produced an altered methylation pattern and an altered sense and antisense RNA transcription of some endogenous REs. We did not find major differences in morphology, in karyotype, or in the expression of pluripotency and differentiation markers, indicating that alterations in retrotransposon methylation and/or expression may be the reason for the problems associated with this late passage ES cell line. We have also observed that in ES cells, in addition of the transcriptional gene silencing by methylation, other post-transcriptional process is involved in gene silencing of some REs. It has recently reported that growth constrained cultures of ES cells are associated with alterations in the methylation pattern of the regulatory domains of imprinted genes leading to altered expression [36]. These observations in conjunction with our results suggest a role for particular epigenetic factors in the loss of ES cell developmental potential.

The best documented mechanism to guard against harmful genomic consequences of REs activation is the transcription gene silencing by DNA methylation of promoters, to impede access of transcription factors or lead to an inactive form of chromatin at target loci [37]. However, it is unlikely that transcriptional silencing can prevent activity of all REs, and other mechanism like RNAi has been described in early stages of development [18]. The relationship between the reactivation of IAP retrotransposon mRNA expression and the demethylated of R1p27 ES cell genomes confirmed that cytosine methylation has an essential role in the suppression of retrotransposons in mammalian stem cells, and agree with previous reports in other cells types where its expression is repressed also by DNA methylation [38]. However, the high expression of MuERV-L retrotransposon in R1p27 ES cells is not related with a reduction in methylation, indicating that, in addition to the methylation control of retrotransposon expression, other mechanisms (i.e. RNAi) may constrain the expression of some repetitive parasitic sequences in ES cells. In agreement with our results, it has been reported that methylation is not the only factor determining endogenous transcriptional activity of REs in ES cell [10]. In addition, in has been reported that the loss of Dicer (the nuclease that initiates RNAi) in ES cells compromises their proliferation, indicating that the RNAi machinery is essential for ES cell proliferation [39]. Also, Dicer knockout mouse ES cells exhibit increased transcription from some repeat sequences, like IAP, combined with severe developmental defects [40]. Our results confirm that in addition of the transcriptional gene silencing by methylation, other post-transcriptional processes are involved in gene silencing of some REs. The fact that the methylation on MuERV-L was not affected in these R1p27 cells, and that the expression of some transcriptional factors, such as, Oct-4 and Nanog, whose expression is correlated with the DNA methylation status in ES cells [41] was also not affected was not surprising, since the differential hypomethylation of different regions of the ES cell genome has been recently reported in mice [42].

Endogenous retrovirus-like (ERV) sequences cover approximately 10% of the mouse genome and 8% of the human genome [37]. Mouse ERVs are active, accounting for 10–15% of all spontaneous insertional mutations in mouse and contributing to numerous cases of cancer [37]. It has been recently shown that human L1 retrotransposon generates a somatic variation which influences both gene expression and cell differentiation [11]. DNA methylation is thought to have evolved as a genome defence mechanism and to have acquired a role in genome regulation during development [43]. The analysis of IAP elements in our ES cell lines suggests that the aberrant methylation pattern of IAP could be responsible for the kinky tail phenotype observed in chimeric mice. In agreement with our hypothesis is the variable phenotype that we have found in our chimeras, which may be a consequence of the stochastic nature of the establishment of methylation marks. There are other examples where epigenetic events have been associated with the kinky tail phenotype. It has been seen in mutants, involving imprinted genes like the IGF2 receptor [44], or the T-associated maternal effect locus [45]. Another example of kinky tail occurrence, where the phenotypic variation cannot be explained by genetic or environmental heterogeneity, is associated with the murine *axin fused (Axin*^*Fu*^) mutant allele, where the presence or absence of this characteristic phenotype, correlates with differential DNA methylation at an IAP retrotransposon [46]. The phenotype is variably expressed among individuals, and in some mice the tail appears completely normal. The stochastic nature of the establishment of the epigenetic state of the 5'LTR portion of the IAP retrotransposon within *Axin*^*Fu*^, leads to the variable expression of the adjacent coding exons among isogenetic littermates [46]. Moreover, it has been observed that in *Axin*^*Fu *^mice that have lost the mutation (IAP is absent and *Axin*^*Fu *^has reverted to the wild allele), there is a spontaneous reappearance of the mutation associated with restoration of the IAP insertion [47]. In mammals, a number of mutant alleles associated with the insertion of IAP retrotransposons have been identified, and it has been observed that the expression of these alleles is affected by the activity and methylation marking of the inserted retrotransposon. IAP retrotransposons may have both upstream and downstream effects on transcription at the site of insertion [48]. The *agouti viable yellow (A*^*vy*^*), agouti hypervariable yellow (Ahvy), agouti intra-cisternal A particle yellow (Aiapy), CDK5 activator-binding protein-IAP*, and the previously mentioned *Axin*^*Fu*^, are examples of such mutant alleles. These alleles have been termed metastable epialleles because they show a variety of unusual characteristics, including the variable expression among genetically identical individuals [49]. Moreover, it has been observed the transgenerational inheritance of some of them (i.e. *Axin*^*Fu*^*and A*^*vy*^), in consequence of hypermethylation within the LTR at the 3'end of an IAP element [46].

It has been reported that the epigenetic alteration that arises in ES cells as a consequence of derivation and culture, is not corrected during postimplantation development, becoming associated with aberrant imprinted gene expression in the foetus [50]. As previously mentioned, in mammals, DNA methylation has a key role in the regulation of the ES cells genome [43]. From the results of this study and the available evidence, we believe that the epigenetic mechanism associated with the kinky tail phenotype observed in our chimeras is associated with the incorrect methylation of the active IAP element present in our R1p27 ES cell line. Incorrect methylation may be also responsible for the alteration in mRNA expression detected in R1p27 ES cells for some of the markers of pluripotency and differentiation analysed. In relation to this, we would like to point out that the abnormal expression of Genesis in these cells may be irrelevant, since it has been demonstrated that this transcript is also absent in human stem cells [24] and probably not essential for pluripotency.

## Conclusion

Our study indicates that epigenetic alterations of some endogenous retrovirus-like might occur in the genome of ES cells and may be responsible for unexpected phenotypes, such as kinky tail, in the chimeric animals produced with them. A second consideration is that in addition of the transcriptional gene silencing by methylation, other post-transcriptional processes are involved in gene silencing of some REs in ES cells. A third consideration is that inadvertent epigenetic instability would have important consequences for the use of ES cells in cell therapy; because REs may induce de novo germ line mutation, are frequent mutagens in some tumours, and can activate oncogenes or cytokine genes [37]. The epigenetic stability of these elements in any ES cell line must be analyzed before their therapeutic use. A fourth consideration is the suggestion that the analysis of REs may be an easy way of evaluating the epigenetic stability of a particular ES cell line. Finally, our results may have implications on the selection and design of retroviral vectors for ES cell gene therapy, necessary to avoid sense and antisence interference phenomena.

## Authors' contributions

MAR conducted ES cells culture procedures and chimera production. EP performed molecular analysis. RFG performed the methylation analysis. PM participated in the chimera production. BP participated in the conception of the study and critically reviewed the manuscript. AGA designed the study and drafted the manuscript. All authors read and approved the final manuscript.
